# Evaluation of Self-Field Effects in Magnetometers Based on Meander-Shaped Arrays of Josephson Junctions or SQUIDs Connected in Series †

**DOI:** 10.3390/mi12121588

**Published:** 2021-12-20

**Authors:** Denis Crété, Julien Kermorvant, Yves Lemaître, Bruno Marcilhac, Salvatore Mesoraca, Juan Trastoy, Christian Ulysse

**Affiliations:** 1 Unité Mixte de Physique CNRS/THALES, Université de Paris-Saclay, CEDEX, 91720 Palaiseau, France; yves.lemaitre@cnrs-thales.fr (Y.L.); bruno.marcilhac@cnrs-thales.fr (B.M.); salvatore.mesoraca@thalesgroup.com (S.M.); juan.trastoy@thalesgroup.com (J.T.); 2 THALES SIX, 92230 Gennevilliers, France; julien.kermorvant@thalesgroup.com; 3 Centre de Nanosciences et de Nanotechnologie, CNRS, 91120 Orsay, France

**Keywords:** Josephson junction, superconducting quantum interference devices, arrays, self-field effect, magnetometer, dynamic, 85.25.-j, 07.07.Df, 07.55.Ge

## Abstract

Arrays of superconducting quantum interference devices (SQUIDs) are highly sensitive magnetometers that can operate without a flux-locked loop, as opposed to single SQUID magnetometers. They have no source of ambiguity and benefit from a larger bandwidth. They can be used to measure absolute magnetic fields with a dynamic range scaling as the number of SQUIDs they contain. A very common arrangement for a series array of SQUIDs is with meanders as it uses the substrate area efficiently. As for most layouts with long arrays, this layout breaks the symmetry required for the elimination of adverse self-field effects. We investigate the scaling behavior of series arrays of SQUIDs, taking into account the self-field generated by the bias current flowing along the meander. We propose a design for the partial compensation of this self-field. In addition, we provide a comparison with the case of series arrays of long Josephson junctions, using the Fraunhofer pattern for applications in magnetometry. We find that compensation is required for arrays of the larger size and that, depending on the technology, arrays of long Josephson junctions may have better performance than arrays of SQUIDs.

## 1. Introduction

Superconductive quantum interference devices (SQUIDs) have excellent performance in magnetometry, offering simultaneously very high transfer factors, very low noise and very large bandwidth [1]. Using them in a flux-locked loop [2] adds the advantage of a large dynamic range. Moreover, developments in noise reduction techniques have become standard practice [3,4,5], reaching noise levels of approximately 0.1 fT/*√*Hz for low-critical-temperature (LTc) SQUIDs [6,7] and less than 10 fT/*√*Hz for high-critical-temperature (HTc) SQUIDs [8,9,10,11,12], depending on the probing loop size [13,14]. These techniques can be used for applications that do not require bandwidth in excess of a few MHz (or 150 MHz when only the flux-locked loop technique is used). These limitations are due either to the size of the feedback loop or to the speed of the modulation electronics. In addition, the periodic response of the SQUID introduces ambiguity [15] corresponding to an integer number of flux quanta $\Phi_{0}=h/2e$, where *h* is Planck’s constant and *e* is the electron charge. In the case of loop unlocking, when the locking point is recovered, it is difficult—if not impossible—to infer the number of flux quanta introduced in the SQUID. Schönau et al. [16] proposed a multi-SQUID architecture to solve this issue. It involves a few SQUIDs with different field periodicity and a data processing unit to decode the value of the magnetic field. The larger the targeted dynamic range, the larger the number of SQUIDs, and the more complex is the processing unit, thereby reducing the bandwidth. For application at a higher frequency, it is necessary to reduce the loop size or to eliminate it altogether. Cold FLL electronics allow loop size reduction and increase the bandwidth up to 350 MHz [17]. Note that two-stage amplifiers can operate at much higher frequencies [18,19,20], but the feedback is not designed for flux-locked operation, so their dynamic range is limited. Using arrays of SQUIDs is an alternative approach allowing an increase in the dynamic range, without drastic effects on the speed [21,22,23,24,25]. Of course, the length of the array must be smaller than the wavelength. Generally speaking, the resulting arrays can be considered 2D arrays of Josephson junctions (JJs). Lefebvre et al. also proposed one-dimensional arrays of JJs connected in series with the advantage of a very large dynamic range [26]. The aim of this paper is to evaluate the potential of arrays of SQUIDs and 1D arrays of JJs taking into account size effects and dispersion of JJ parameters for the same estate on the substrate. For the sake of simplicity, we assume that all the SQUIDs (and/or JJs) are identical. In Section 2, we recall the principle of magnetic field detection and the ideal scaling of the performance with the number of JJs in an array. In Section 3, we introduce self-field effects, distinguishing between intra-SQUID and inter-SQUID origins; their effects are evaluated in Section 4, first for the impact of layout, and then for the impact of JJ dispersion, which is especially relevant for the HTc technology, to evaluate the scaling of series arrays of SQUIDs for large *N*. In Section 5, we propose a solution to compensate for the inter-SQUID self-field effects. In Section 6, the same evaluation is made for series arrays of HTc Josephson junctions. Finally, the results obtained in Section 4 and Section 6 are compared in Section 7, which shows that, for the average level of HTc JJ dispersion, series arrays of JJs may challenge series arrays of SQUIDs, with the advantage of a much larger dynamic range. In the conclusions, we summarize the assumptions and results presented, with a focus on RF detection.

## 2. Ideal Devices

### 2.1. Single SQUID

**Figure 1:**
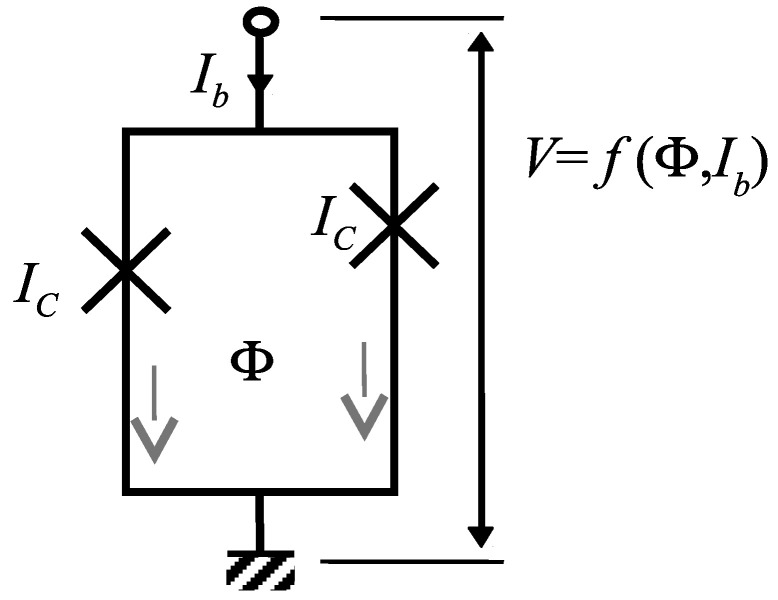
Basic SQUID with perfect symmetry, with crosses representing JJs. The magnetic flux originating from the bias current has zero net value.

The ideal SQUID is made with two identical JJs, connected in parallel in order to form a superconducting loop, as illustrated in Figure 1. This loop is characterized by a loop inductance $L_{S}$, relating the magnetic flux Φ to the current circulating around the loop, i.e., half the difference in the currents $i_{1}$ and $i_{2}$ flowing in each JJ:

(1) $$\Phi =L_{S}\frac{i_{1}-i_{2}}{2}.$$

Because of the first Josephson relation [27], the JJs act as detectors of superconducting wave function phase difference (WPD) between their electrodes. In the absence of magnetic flux density (B=0), and provided that the SQUID is symmetric, i.e., not only the JJs but also the branches are identical, then the WPDs of each JJ are equal. In this case, their currents $i_{1}$ and $i_{2}$ are equal and the magnetic flux Φ is 0. Assuming a strong coupling between the JJs (low-inductance SQUID), the SQUID behaves as a single JJ with critical current $2I_{C}$, where $I_{C}$ is the critical current of the JJs. The application of a magnetic field with a non-zero component along the normal to the SQUID loop generates screening currents in the electrodes, associated with phase gradients. These phase gradients combine to apply different WPDs on the JJs, and the SQUID behaves as a single JJ with a reduced critical current. Thus, the magnetic field induces a periodic change in the voltage vs. current characteristic ($V_{S}\left(I\right)$) of the SQUID corresponding to 2π rad WPD variation.

### 2.2. Arrays of Josephson Junctions

Table 1 presents the scaling expected for 1D and 2D arrays with the number *N* (resp. *M*) of JJs connected in series (resp. in parallel). This paper evaluates series arrays of JJs (JSA) and/or SQUIDs (SSA). However, the comparison between JSA (*M* = 1) and SSA (*M* = 2) cannot be based only on this table. Since the connection of dipoles in series corresponds to the addition of individual voltages, the output voltage amplitude $V_{N}$ of an array of *N* SQUIDs (resp. JJ) connected in series is *N* times larger than for a single SQUID (resp. JJ), as illustrated in Table 1. Similarly, for SQUIDs, in parallel arrays, the loop inductance must be taken into consideration. The inductance of the largest loop, $L_{A}$ scales approximately as $M\cdot L_{S}$, where $L_{S}$ is the inductance of the elementary cell. Assuming that the elementary loop inductance $L_{S}$ is such that $\beta_{L}\approx 1$, we postulate that the amplitude of the modulation is decreasing with inductance in a comparable way as for a SQUID. Thus, the transfer factor is approximately independent of *M*. Note that the transfer factor would also be independent of *M* for a scaling rule such that the physical width of the array is kept constant. Among 2D arrays, we consider only those arrays obtained by series connection of 1D parallel arrays of JJs. The input noise spectral density $S_{B}$ is defined by the following relation:

(2) $$S_{B}=\frac{S_{V}}{{\left(\frac{\partial V_{N}}{\partial B}\right)}^{2}}.$$

The dynamic range is the ratio of (i) the input power necessary to raise the contribution of non-linearity to a level with a predefined signal-to-noise ratio, which depends on the application; (ii) the input power necessary to raise the contribution of the linear response to the same level with a predefined signal-to-noise ratio. The SFDR is obtained in the same way, but for a signal-to-noise ratio of unity. Ideally, there are a number of parameters that improve when increasing *N*. In particular,

- the input noise spectral density (NSD) $|S_{B}|$, assuming that the SQUID noise contributions are not correlated;
- the spur-free dynamic range (SFDR);
- and the dynamic range, as the output noise is independent of *N*.

Several publications have reported on the experimental observation of this scaling behavior [22,23]. While the results are in good agreement with the theory for the LTc technology, there is a significant discrepancy for the HTc technology, reducing the benefit of using series arrays. In the best case, it may be possible to use larger values of *N* to compensate for this reduction. Otherwise, this will quickly reach a limit because of the impedance of the device, or because of size with respect to wavelength. A parallel arrangement of several JJs will also have advantages thanks to the quantum coherence of superconductors [28,29]. It can be considered as an intermediate geometry between a long JJ and a SQUID with a (large) loop delimited by the two JJs located at each end of the 1D array. The output voltage modulation amplitude is the same as for a single SQUID, provided that the screening parameter 2$L_{A}I_{C}/\Phi_{0}$ is not large compared to unity, i.e., as soon as the array becomes too wide (typically with around 10 JJs, or around 100 μm) [30]. This also results in non-uniform bias current distribution. Finally, 2D arrays made of a series connection of 1D parallel arrays of JJs combine the advantages of both 1D series and 1D parallel arrays, with the additional advantage in the choice of the resulting impedance and total length of the device, which is important for high-frequency applications [31]. The sensitivity (input NSD) improves as the reciprocal of the total number of JJs.

**Figure 2:**
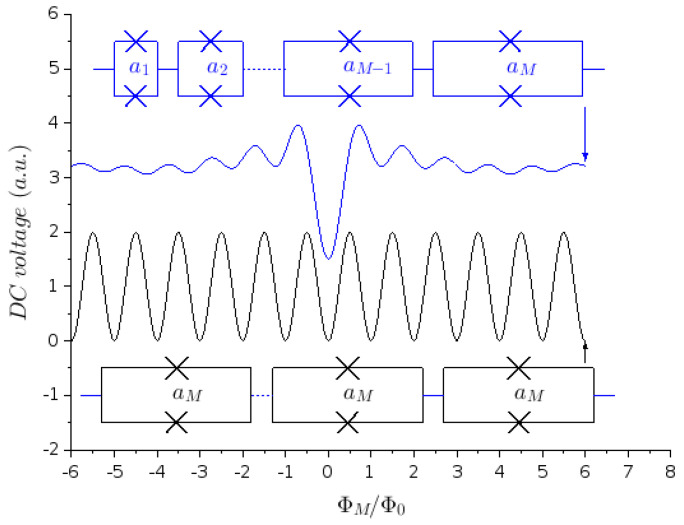
Transfer function made with series arrays of SQUIDs. Top curve: non-periodic with incommensurate SQUID areas; bottom curve: periodic with equal SQUID areas ($\Phi_{M}$ refers to the flux coupled to the SQUIDs with area $a_{M}$). The curves are shifted vertically for clarity.

As suggested by Carelli et al. [24], series arrays of SQUIDs with different effective areas are absolute magnetometers. They can have a non-periodic response provided that the effective areas are incommensurate, as illustrated in Figure 2. Shaping the transfer function is possible but not sufficient to substantially improve linearity. Kornev et al. [29] obtained high linearity with differential architectures involving parallel LTS JJ arrays.

## 3. Self-Flux

### 3.1. Origin

**Figure 3:**
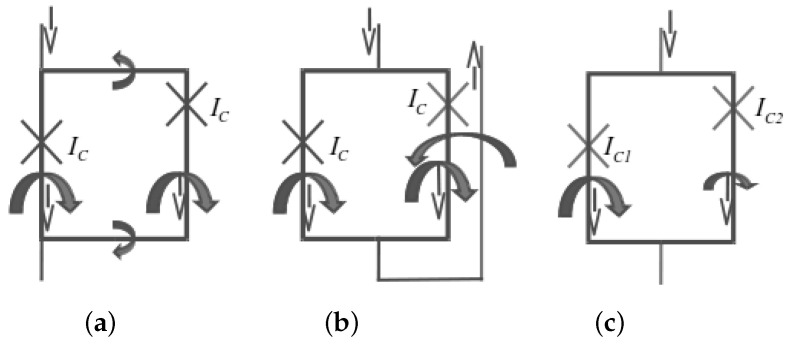
Illustration of the SQUID categories according to symmetry: (**a**) Layout asymmetry in the SQUID loop. (**b**) Layout asymmetry in a bias line. (**c**) Josephson asymmetry (provided $I_{C1}\neq I_{C2}$).

Because the currents circulating in the vicinity of the SQUIDs generate a magnetic field, the resulting local field is not strictly proportional to the applied magnetic field. We assume here that the only current circulating in the vicinity is the bias current *I*. We may distinguish between inter-SQUID effects, where the bias current circulating in a SQUID (or in a nearby bias line) affects another SQUID, and intra-SQUID effects, where the magnetic field generated by a SQUID is sensed by the very same SQUID. In the first case—cf. Figure 3b—the magnetic field originates from the layout only, and in general, a careful design may reduce this contribution, as presented in Section 5. In the second case, the net intra-SQUID flux is associated with SQUID asymmetry resulting from layout asymmetry—cf. Figure 3a—or from JJ asymmetry—cf. Figure 3c. The effect of stochastic deviations of the JJ properties on the SQUID they belong to is evaluated in Section 4.2. On the other SQUIDs, these stochastic deviations have a second-order effect that we neglect in this study.

### 3.2. Layout Asymmetry

In the case of layout asymmetry, as illustrated in Figure 3a,b, the evaluation of the self-flux requires only the integration of the Biot–Savart law over the effective area of the SQUID. This asymmetry can be introduced by design [32] to increase the transfer factor of a single SQUID magnetometer. When the asymmetry results from stochastic deviations due to the microfabrication process, its effect is expected to be negligible. Indeed, the systematic errors, e.g., in photoresist mask edge positions or in layer over-etching, do not break the symmetry of the circuit to first order and their stochastic deviations are averaged over the size of the SQUID loop, i.e., much larger than their correlation size. It is then essentially a matter of design to control the contribution of asymmetry. The expression for the intra-SQUID source is given by [33]:

(3) $$\Phi_{1a}=\left(L_{R}-L_{L}\right)\frac{I}{2},$$

where $L_{R}$ and $L_{L}$ are the inductances of the right and left branches of the SQUID in case *a* and

(4) $$\Phi_{1b}=M_{S}\cdot I,$$

where $M_{S}$ is the mutual inductance of the bias line and the SQUID in case *b*.

**Figure 4:**
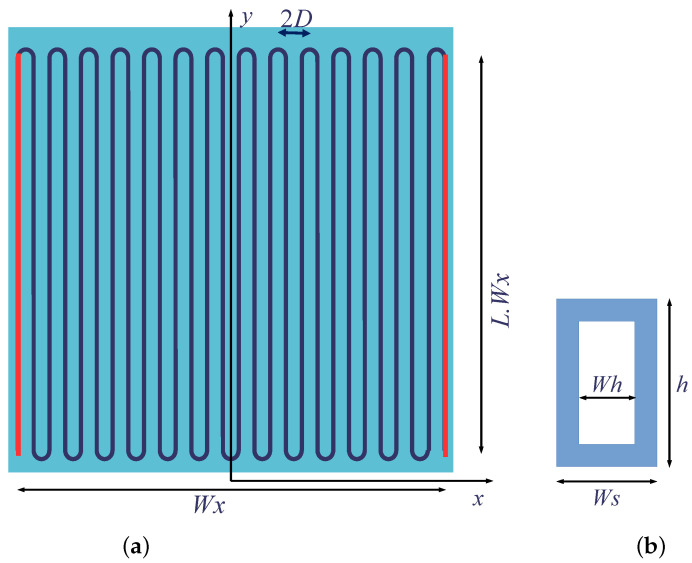
Schematic representation (**a**) of the meander geometry, with *J* = 28 including the edge segments represented in red; (**b**) of the SQUID geometry (JJs are not represented).

**Figure 5:**
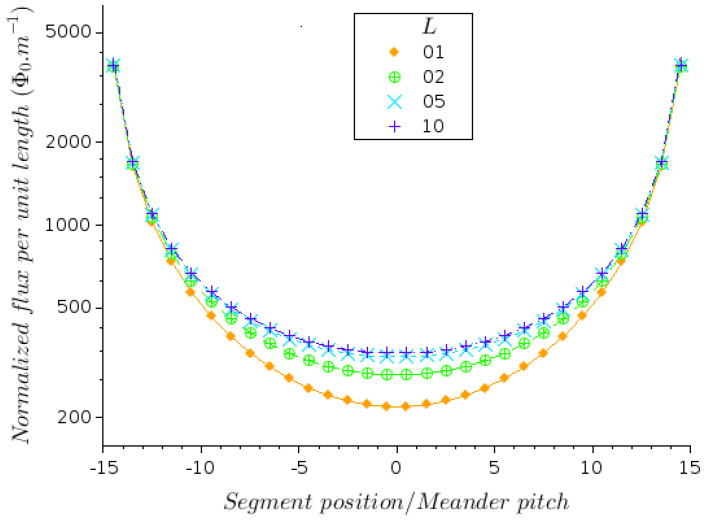
Flux distribution over the SQUIDs located at the center of each segment of the meander geometry for a bias current I=100μA and different segment lengths $W_{y}=L\cdot W_{x}$. The symbols are located at the position of the center of each segment. The flux per unit length is given in $\Phi_{0}/m$.

To illustrate the contribution of the inter-SQUID source in case *b*, we take the example of a 1D series array of a large number of SQUIDs. The array is laid out on a rectangular substrate and is folded in a meander configuration, as represented in Figure 4a. Due to the symmetry of the device, the self-flux in the center is minimal. It vanishes on the segment that may happen to be on the axis of symmetry (i.e., in the case of an odd number of segments). On the outermost segments, the self-flux reaches a maximum. Figure 5 illustrates the flux distribution on the segments of the array. As this difference in self-fluxes corresponds to a phase shift between the SQUIDs, it contributes to the degradation of the overall response to a magnetic field.

### 3.3. Evaluation of Self-Flux in a Meander Arrangement

Our model assumes that the meander is made up of *J* straight segments repeated with a pitch *D*, as shown in Figure 4a. Most (or all) of these segments contain SQUIDs connected in series. Thus, the overall dimension in the *x* direction is $W_{x}=\left(J-1\right)\cdot D+W_{s}$, where $W_{s}$ is the width of the SQUID (cf. Figure 4b). This length is used to normalize the segment length $W_{y}=L\cdot W_{x}$. The SQUID extension in the *y* direction is *h*. A semi-circular track connects the ends of consecutive segments in the series array. A current running in meander line *k* generates a magnetic field $B^{k}$. The magnetic field is calculated at each point (*x*, *y*) as the field resulting from the current flowing in each segment.

(5) $$B^{k}=\frac{\mu_{0}\cdot I}{4\pi }\int \;\frac{dy_{k}j\times \left(\left(x_{k}-x\right)i+\left(y_{k}-y\right)j\right)}{{\left({\left(x_{k}-x\right)}^{2}+{\left(y_{k}-y\right)}^{2}\right)}^{3/2}},$$

where $\mu_{0}$ is the magnetic permeability; **i** and **j** are unit vectors along the *x* and *y*-axes. The total magnetic field on segment *i* is:

(6) $$B_{z}\left(x,y\right)=\sum_{k\neq i}B_{z}^{k}\left(x,y\right)=\sum_{k\neq i}{\left(-1\right)}^{k}\frac{\mu_{0}\cdot I}{4\pi (x_{k}-x)}{\left[\frac{y_{k}-y}{\sqrt{{\left(x_{k}-x\right)}^{2}+{\left(y_{k}-y\right)}^{2}}}\right]}_{-W_{y}/2}^{W_{y}/2}.$$

In summation, the contribution for k=i is either negligible, or considered to be an intra-SQUID contribution evaluated in Section 3.4. We neglect the magnetic field created by the semi-circle connections at the ends of the segments as a higher-order effect—and even changing sign in the central part of the device (y=0). Then, the distribution of the SQUIDs along the segments is defined and the self-flux calculated. The dimensions $W_{x}$ and $W_{y}$ can be regarded as the substrate dimensions. We evaluate the self-flux per unit length $\phi^{s}$ by integration over the effective width of the SQUID along the *x* direction, i.e., between $x_{i}^{-}=x_{i}-\left(W_{s}+W_{h}\right)/2$ and $x_{i}^{+}=x_{i}+\left(W_{s}+W_{h}\right)/2$ for segment number *i*. We choose to compare several SQUID distributions with the same device width $W_{x}$, and for different values of the current $I_{e}$ flowing in the edge segments. SQUID distributions differ by the segment length $W_{y}$ and by the SQUID density (or equivalently by their *y*-dimension *h*).

(7) $$\phi^{s}\left(x_{i},y\right)=\int_{x_{i}^{-}}^{x_{i}^{+}}\;B_{z}\left(x,y\right)\;dx$$

We note that the self-flux is minimal in the center, and it would be zero for an odd number of segments in the device. The self-flux is the maximum for the outermost segments. Note that the self-flux will be larger for larger bias currents, i.e., for 2D arrays (even though the pitch *D* along the *y* direction will be larger, due to the larger width of the segments). Moreover, the maximum absolute value on the edges oscillates with *J* in a similar way as for infinitely long segments, i.e., $|\sum_{k=1}^{J}\frac{{\left(-1\right)}^{k}}{k}|$ around the asymptotic value log(2). It means that (a) this maximum is the largest for J=4; (b) for large *J*, the field on the edges depends only weakly on *J*. The main factors governing the field amplitude on the edges are the bias current and the pitch of the meander. To put this in a more quantitative form, we note that in Equation (6), the contributions of k=i−1 and k=i+1 cancel each other out. Thus, the number of terms entering the summation can be reduced. In the case of an even number of segments, $B_{z}$ is an even function and we can restrict the calculation to i≤J/2. For y=0, the magnetic field at the center of a SQUID is given by the following expression:

(8) $$B_{z}\left(x_{i},0\right)=\frac{\mu_{0}I}{2\pi D}\sum_{k=2i}^{J}\frac{{\left(-1\right)}^{k}a}{\left(k-i\right)\sqrt{{\left(k-i\right)}^{2}+a^{2}}}$$

where $a=W_{y}/2D$. As seen in Figure 5, the field is the largest on the edges, where it is almost independent of *a* when $W_{y}>>D$. To evaluate Equation (8), we separate the first term (for k=2i) and group the remaining J−2i terms 2 by 2 to emphasize that their contribution is small. After approximating the summation by a continuous integral, we obtain the magnetic field at y=0:

(9) $$B_{z}\left(x_{i},0\right)\approx \frac{\mu_{0}I}{2\pi D}\left[\frac{a}{i\sqrt{i^{2}+a^{2}}}+\frac{1}{2}log\left(\frac{f(i+2)f(J-i-1)}{f(i+1)f(J-i)}\right)\right]$$

where $f\left(u\right)=a/u+\sqrt{{\left(a/u\right)}^{2}+1}$. As expected, when the number of segments *J* is large, the *z*-component of the field distribution on the central SQUIDs depends essentially on *a* and the prefactor I/D.

### 3.4. Josephson Asymmetry

The JJ asymmetry (Figure 3c) may result from design as well, or from stochastic deviations of JJ parameters (dispersion). In both cases, the self-flux is given by [33]:

(10) $$\Phi_{2}=L_{S}\cdot \Delta I_{C}$$

where $L_{S}$ is the loop inductance of the SQUID, and $\Delta I_{C}$ is the difference in the critical currents of the JJs. The resulting self-flux $\Phi^{sf}$ is the sum of $\Phi_{1}$ and $\Phi_{2}$:

(11) $$\Phi^{sf}=\left(M_{S}+L_{R}-L_{L}\right)\cdot I+L_{S}\cdot \Delta I_{C}.$$

We reported experimental results on single SQUIDs illustrating the evolution of self-flux with the bias current and the temperature in [34].

## 4. Evaluation of Self-Flux Degradation on the Array Performance

### 4.1. Impact of Layout

As we explore different sizes of the SQUID, it is necessary to account for the variation in the SQUID inductance $L_{s}$. Given the geometry of Figure 4b, with D=13μm, $W_{h}$ = 5 μm, $W_{s}=9\;\mu$m and *h* is a free parameter, we used InductEx [35] to evaluate the inductance as a function of *h* and fitted the results with a least square second-order regression to obtain

(12) $$L_{S}=-0.5422+1.6529\cdot h-0.008787\cdot h^{2}$$

where $L_{S}$ is in picohenry and *h* in micrometers. For a single SQUID, the modulation voltage is $\Delta V_{S}=\Delta V_{0}/\left(1+\beta_{L}\right)$, where $\beta_{L}=2L_{S}I_{C}/\Phi_{0}$ and $\Delta V_{0}$ is essentially the $I_{C}R_{n}$ product as we can neglect the correction for thermal noise effect proposed by Enpuku et al. [36]. We assume that the SQUID critical current 2$I_{C}$ is equal to the bias current. The accuracy required is not drastic for the case of high-inductance SQUIDs, and it is even less for the smaller SQUIDs. Thus, the voltage modulation $\Delta V_{N}$ of a series array of *N* SQUIDs is:

(13) $$\Delta V_{N}=\sum_{k=1}^{N}\frac{V_{0}\cdot cos\left(\frac{2\pi }{\Phi_{0}}\left(\Phi^{a}+h\phi^{sf}\left(x_{i},y_{j}\right)\right)\right)}{1+\beta_{L}}$$

where SQUID *k* is the *j*-th SQUID of segment *i*; $\Phi^{a}$ is the applied flux, assumed to be uniformly distributed for the (identical) SQUIDs, and $\phi^{sf}=\Phi^{sf}/h$ (cf. Equation (11)), which is equal to $\phi^{s}$ if the SQUIDs are symmetric as in Figure 1. We note that the self-field effect due to the meander layout is small for bias currents smaller than ≈1 mA. Thus, the result of the numerical model can be summarized for 6<h<16μm by the following expression for the maximum of the transfer function:

(14) $$\frac{\partial V_{N}}{\partial B_{a}}=\frac{\partial V_{0}}{\partial \Phi }\frac{W_{x}W_{y}W_{S}}{D(1+C/N)}$$

where $V_{0}$ is the voltage across a single low-inductance SQUID, and keeping only the linear term in $L_{S}\left(h\right)$, in Equation (12),

(15) $$C\approx 1.65\cdot 10^{-6}\frac{W_{y}W_{x}}{D}\cdot \frac{2I_{C}}{\Phi_{0}}.$$

where the dimensions are all in meters. With a square footprint on the substrate (*L* = 1), 2$I_{C}=200\;\mu$A and the meander geometry described above, C≈1600.

### 4.2. Impact of Scattering

The dispersion of JJ characteristic parameters translates to Josephson asymmetry. We have carried out simulations of the effect of critical current dispersion for the case of a series array of *N* = 2000 SQUIDs with identical loops. The model is further simplified, noting that, for series arrays of SQUIDs, the amplitude of each harmonic of the array voltage response is the sum of the harmonic amplitude for all the SQUIDs. As the phase fluctuations of the harmonic components are linearly increasing with the harmonic order, harmonic components will be more sensitive to SQUID asymmetry. Phase shifts will prevent a large amplitude of the resulting spectral components. Thus, it is sufficient to model the SQUID responses using sinusoidal transfer functions. Typically, the voltage modulation amplitude of the SQUID array, $\Delta V_{N}$, decreases exponentially until it saturates, when the phase shifts approach uniform distribution, i.e.,

(16) $$\Delta V_{N}\approx \sqrt{N}\cdot \Delta V_{S},$$

where $\Delta V_{S}$ is the voltage modulation for a single SQUID. The SQUIDs are not contributing constructively and their contribution scales as $\sqrt{N}$, as do the noise contributions: there is no advantage for applications. Before saturation, i.e., for smaller dispersion, the voltage modulation amplitude $\Delta V_{N}$ can be fitted by the following expression [37]:

(17) $$\Delta V_{N}\approx N\cdot \Delta V_{S}\cdot e^{-2\sigma_{I}^{2}{\left(\frac{\pi L_{S}}{\Phi_{0}}\right)}^{2}}$$

where $\sigma_{I}$ is the standard deviation for the critical currents. We define the degradation as the ratio of $\Delta V_{N}$ and the ideal scaling:

(18) $$\frac{\Delta V_{N}}{N\cdot \Delta V_{S}}\approx e^{-2{\left(2\pi \beta_{L}\cdot \frac{\sigma_{I}}{I_{C}}\right)}^{2}},$$

Assuming $\beta_{L}=1$ and $\sigma_{I}/I_{C}=13\%$ gives a 3dB degradation compared to ideal scaling. This degradation is much larger for larger values of $\sigma_{\phi }=4\pi \beta_{L}\sigma_{I}/I_{C}$, the standard deviation of the “phase-shift” resulting from the self-field due to critical current dispersion. Taking into account a dispersion in the normal resistance as well does not drastically change the results.

## 5. Compensation

Attempting to compensate for meander self-flux by using intentional asymmetric SQUIDs is possible to a very limited extent: the self-flux for asymmetric bias contact is limited by $L_{S}\cdot I_{C}$, which we want to keep less than $\Phi_{0}$, or even lower for HTc technologies [38]. For the same reason, using the Josephson asymmetry is probably even less efficient.

**Figure 6:**
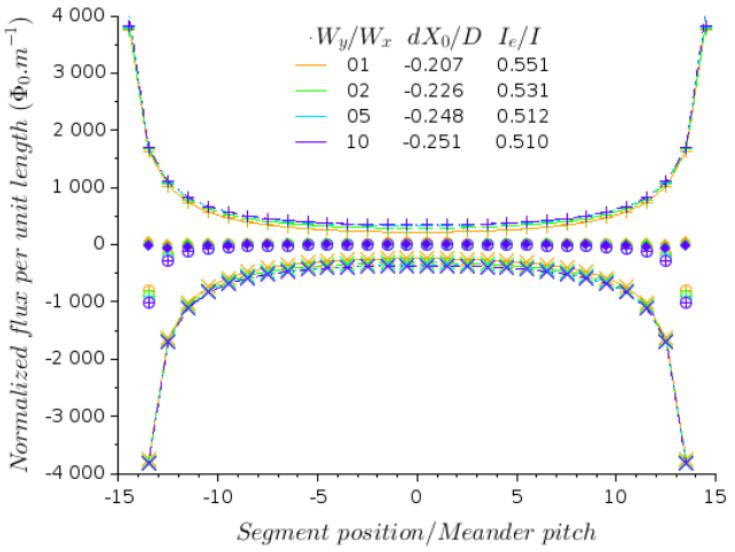
Flux distribution over the SQUIDs located at the center of each segment of the meander geometry for a bias current I=100μA. The flux per unit length is normalized by the flux quantum $\Phi_{0}$. The symbols refer to different configurations of the edge segments: (+) containing SQUIDs as all the other segments (same as Figure 5); (×) no SQUID on edge segments, and $I_{e}=0$; (⊕) no SQUID on edge segments, and $I_{e}$ as indicated in the table as inset; (⋄) idem, but edge segments are shifted by $dX_{0}$ as indicated in the table as inset.

From Figure 6, we note that for an even number of segments *J*, the self-flux changes sign with the parity of J/2. This indicates that replacing a pair of segments—one at each edge—by striplines, where around half of the bias current *I* is flowing, will drastically reduce the amount of self-flux in all the SQUIDs. This is optimized by the fit proposed in Equation (19):

(19) $$f\left(x_{i}\right)=\Phi_{A}\cdot \left(\frac{1}{x_{i}-X_{0}}+\frac{1}{x_{i}+X_{0}}\right)+\Phi_{B},$$

representing two contributions: (i) a compensation field created by two segments placed at $+X_{0}$ and $-X_{0}$ (in red in Figure 4) and fed with a current proportional to $\Phi_{A}$; (ii) a uniform offset $\Phi_{B}$, which is not critical as it may easily be changed by application of the “bias field”, i.e., the field that is necessary to operate the array at the best point. The result is shown in Figure 6: the plain line joining the symbols “×” represents the fit to self-flux when the bias current flows only in the inner “segments”, and not in the edge segments. The table contained in this figure reports $dX_{0}/D$ defined as the normalized displacement of the edge segments from their original position (i.e., the distance separating the edge segments is changed by 2$dX_{0}$), and $I_{e}/I$ the fraction of the bias current for optimal compensation. The latter value is close to 0.5, as is expected from the curves, with “+” and “×”, respectively, corresponding to $I_{e}/I=1$ and $I_{e}/I=0$. The set of data reported by “⊕” symbols are obtained for edge segments maintained at their original position, but fed with the current $I_{e}$ as in the table. Finally, the set of data reported by “⋄” symbols are obtained for edge segments slightly shifted from their original positions towards the center of the device (while keeping the central symmetry), and fed with the current $I_{e}$ as in the previous case. This indicates that an excellent compensation for the self-field can be achieved provided that the extra segments are properly located and electrically fed. After compensation, the residual flux can be less than approximately 1% of the initial self-flux. However, compensation will be more efficient when *J* is even.

With SQUIDs of identical size, although the transfer function (applied magnetic field to voltage conversion factor) is smaller for smaller SQUIDs, they seem to be better for the following reasons:

- it is necessary to keep $\beta_{L}$ small to maintain the modulation amplitude of individual SQUIDs;
- smaller SQUIDs are less sensitive to “inter-SQUID” self-flux;
- and, as seen in Section 4.2, they are less sensitive to “intra-SQUID” self-flux.

Impedance matching is an important criterion when coupling an available power to a load. Here, the available power depends on the number of SQUIDs in series and the load is fixed by the input impedance of the readout electronics $R_{0}$, generally 50 Ω. When increasing the size of the substrate, i.e., the length of the array, the voltage amplitude saturates, with an asymptotic limit value of $V_{\infty }=2R_{0}/R_{1}V_{S}$, where $R_{1}$ is the resistance of the elementary SQUID. If the SQUID noise is not correlated, the noise power of the device scales as $N\cdot N_{1}$, with $N_{1}$ being the noise for a single SQUID. The signal-to-noise ratio is then evaluated taking into account the input noise $N_{A}$ of the readout electronics, and $R_{0}$ is assumed to be real:

(20) $$SNR=\frac{{\left(\delta V_{N}\right)}^{2}}{N_{N}}=\frac{\frac{R_{0}}{2}\cdot {\left(\frac{\frac{\partial V_{N}}{\partial B_{a}}\delta B_{a}}{R_{0}+N\cdot R_{1}}\right)}^{2}}{N_{A}+\frac{R_{0}R_{1}}{2}\cdot \frac{N\cdot N_{1}}{{\left(R_{0}+N\cdot R_{1}\right)}^{2}}}.$$

After separation of the geometrical parameters, we obtain:

(21) $$SNR=\frac{{\delta B_{a}}^{2}}{R_{1}}{\left(\frac{\partial V_{0}}{\partial \Phi }\right)}^{2}\frac{{\left(W_{x}W_{y}W_{S}/D/\left(1+C/N\right)\right)}^{2}}{N\cdot N_{1}+\frac{2N_{A}}{R_{0}R_{1}}\cdot {\left(R_{0}+N\cdot R_{1}\right)}^{2}}.$$

For the geometry considered in Figure 4, and if the amplifier noise dominates, the signal-to-noise ratio is maximum for a value of *N* in the range $R_{0}/R_{1}\ldots C$. It should be taken into account that the SSA noise will reduce the optimal value of *N*, i.e., to use less SQUIDs with larger loops. For the ion-damaged barrier technology, the JJ normal resistance is related to $L_{JJ}$ and typically $\rho =R_{n}L_{JJ}=1\;\Omega \cdot \mu$m. In a SQUID, the JJs are made small, i.e., their length $L_{JJ}=2\;\mu$m. With $R_{0}=50\;\Omega$ and $R_{1}\approx 0.25\;\Omega$, the matching condition is achieved with N≈200. Assuming $\beta_{L}=1$, this corresponds to a substrate size of 20,000 μm${}^{2}$, or a 0.14 mm side square. This SSA can have a transfer factor increased by a factor ≈150 compared to a single SQUID, while being properly matched to RF readout electronics. With a transfer factor of approximately 2 V/T for a small SQUID, this corresponds to a transfer factor of approximately 300 V/T.

Note that this evaluation does not take into account the degradation of SSA performance due to “intra-SQUID” self-flux.

To circumvent the severe limitation on the maximum number of SQUIDs, there are at least three strategies:

- increasing the impedance of the readout electronics, which is possible only for low-frequency devices because it reduces the bandwidth of the system;
- associating devices in parallel to lower its output impedance;
- and using flux transformers and/or flux concentrators [39].

We anticipate that the benefits of this compensation for 2D arrays will be larger, owing to the larger bias current. These 2D arrays are attractive as their impedance can be tailored to match the input impedance of the readout electronics, thereby reducing its noise contribution. For a given array output impedance, the maximum value of *N* scales as *M*: according to Table 1, the sensitivity $S_{B}^{1/2}$ should scale as $M^{-1}$.

In the case of arrays made up of SQUIDs with different sizes, this evaluation indicates that it is preferable to implement the larger SQUIDs in the segments that are close to the center of the device, and the smaller SQUIDs on the edge segments. This arrangement may be sufficient to avoid self-flux degradation and may prove to be better than the implementation of flux compensation as it takes advantage of the whole surface of the substrate for more SQUIDs.

Although combining an SSA of limited size and flux transformer might be a promising approach, this is beyond the scope of this paper.

## 6. Josephson Junction Series Arrays

We now analyze the potential performance of JJ series arrays (JSA) to compare them with “compensated” SQUID arrays, given a substrate area. Using JJs as magnetometers was proposed shortly after the discovery of the Josephson effect [40,41,42,43]. The sensitivity of the JJ to magnetic fields comes from the gradients of the superconducting wave-function phases in each electrode. However, as opposed to the case of the SQUID (cf. Section 2.1), this is now restricted to the region of the barrier, threaded by the magnetic field over a thickness largely dependent on the barrier geometry [44]. Assuming an electrode thickness of around 150 nm, which is less than the London penetration depth $\lambda_{L}$, then, for grain boundary JJs (and for JJs using the ion-damaged barrier technology as well), the effective area $A_{eff}$ is related to the JJ length $L_{JJ}$ by:

(22) $$A_{eff}=0.543\cdot L_{JJ}^{2}$$

For other JJ technologies, this effective area will depend on the distribution of the screening currents. Increasing the barrier length improves, to some extent, the sensitivity, but long Josephson junction effects limit this approach to a junction length $L_{JJ}$ comparable to the Josephson length. Series arrays of Josephson junctions are an interesting approach proposed by Lefebvre et al. [26]. The main advantage of this approach is the non-periodic response to a magnetic field, as there is much less destructive interference of individual responses as for SQUIDs. A potential advantage of the large integration density offered by a few technologies, e.g., the ion-damaged barrier technology [45], is limited by the degradation of the Meissner effect, weakened by the presence of the JJ barriers [37]. Thus, the number of JJs integrated in a given area of the substrate is only slightly dependent on the chosen technology. Reducing the JJ distance $d_{JJ}$ below $\approx L_{JJ}/2$ mostly increases the impedance and noise.

For these technologies obeying Equation (22), we model the flux penetrating the barriers by a simple magnetic dipole. We find the following expression for the effective area of JJ separated by a distance $d_{JJ}$ along the *y*-axis:

(23) $$A_{e}\left(d_{JJ}\right)=\frac{A_{eff}\cdot 2d_{JJ}}{2d_{JJ}+L_{JJ}}=\frac{1.086L_{JJ}^{2}d_{JJ}}{2d_{JJ}+L_{JJ}}$$

The transfer factor of a single JJ is given by

(24) $$\frac{\partial V_{1}}{\partial B_{a}}=A_{e}\left(d_{JJ}\right)\frac{\partial V_{1}}{\partial \Phi }\approx \frac{1.086L_{JJ}^{2}d_{JJ}}{2d_{JJ}+L_{JJ}}\frac{\partial V_{1}}{\partial \Phi }$$

where $B_{a}$ is the macroscopically applied field, and $V_{1}$ is the voltage across a single Josephson junction. As the $I_{C}R_{n}$ product does not depend on $L_{JJ}$, we consider that $V_{1}$ only weakly depends on $L_{JJ}$. Thus, the transfer factor from magnetic flux to voltage for a JJ is essentially determined by the Fraunhofer dependence of the critical current and can be approximated by $1.4I_{C}R_{n}/\Phi_{0}$. In order to estimate the best performance for a given substrate area, we evaluate the achievable dynamic range on a given substrate area, optimizing for $L_{JJ}$, and possibly $d_{JJ}$. We assume a meander layout for the chain of LJJs, with a spacing g=2μm between each straight segment. A device with such a small gap separating the meander segments may require e-beam lithography, but is feasible. We estimate *N*, the number of JJs:

(25) $$N=\frac{W_{x}}{L_{JJ}+g}\cdot \frac{W_{y}}{d_{JJ}}$$

The JSA transfer factor is:

(26) $$\frac{\partial V_{N}}{\partial B_{a}}=N\cdot \frac{\partial V_{1}}{\partial B_{a}}=\frac{1.49W_{x}W_{y}L_{JJ}^{2}}{\left(2d_{JJ}+L_{JJ}\right)\left(L_{JJ}+g\right)}\frac{I_{C}R_{n}}{\Phi_{0}}$$

where $V_{N}$ is the output voltage of the JSA. We use Equation (20) to evaluate the signal-to-noise ratio, where $\partial V_{N}/\partial B_{a}$ is now given by Equation (26), $R_{1}$ is the normal resistance of one LJJ, and *N* is the number of LJJs:

(27) $$SNR=\frac{\delta B_{a}^{2}}{\rho }\cdot {\left(\frac{I_{C}R_{n}}{\Phi_{0}}\right)}^{2}\frac{2.21W_{x}^{2}W_{y}^{2}L_{JJ}^{5}}{\left(NN_{1}+\frac{2N_{A}}{R_{0}R_{1}}\cdot {\left(R_{0}+N\cdot R_{1}\right)}^{2}\right){\left(2d_{JJ}+L_{JJ}\right)}^{2}{\left(L_{JJ}+g\right)}^{2}}.$$

We assume no noise correlation between the JJs, $N_{N}=N\cdot N_{1}$, and that the noise in an LJJ scales as $1/L_{JJ}$[46]: $N_{1}=N_{0}\cdot \left(L_{0}/L_{JJ}\right)$, where $N_{0}$ (resp. $N_{1}$) is the noise power for the reference JJ (resp. the JJ with length $L_{JJ}$). Then, the $L_{JJ}$ dependence can be written as $L_{JJ}^{7}/P_{7}\left(L_{JJ}\right)$, where $P_{7}$ is a polynomial of degree 7 with exclusively positive coefficients: the SNR is a strictly increasing function of $L_{JJ}$ and the maximum value is obtained for the largest technologically feasible $L_{JJ}$. Following Lefebvre et al. [26], we choose a maximum value of $L_{JJ}=2\cdot L_{0}=10\;\mu$m. We observe that the optimal value of $d_{JJ}$ is close to $L_{JJ}/2$, as $L_{JJ}$ is very small compared to the substrate size. Thus, $d_{JJ}$ is $\approx L_{0}$, i.e., well above the limit encountered for the ion-damaged technology, below which the $I_{C}R_{N}$ product degrades [37]. If the amplifier noise power dominates, then the total number of JJs and required substrate area are given by

(28) $$N=\frac{R_{0}L_{JJ}}{\rho }$$

(29) $$A^{2}=\frac{R_{0}L_{JJ}^{2}\left(L_{JJ}+g\right)}{2\rho }$$

With $L_{JJ}=10\;\mu$m and g=2μm, we obtain N≈500, and the substrate area is $3\times 10^{-8}$ m${}^{2}$, e.g., a 170μm side square substrate. The transfer factor of the array is ≈500 times larger than for a single junction, and should reach approximately 1000 V/T.

## 7. Discussion

We evaluated transfer factors for SSA and JSA, in the latter case without taking into account the self-field effects. We have shown in Section 5 that we can compensate for the inter-SQUID self-field in meandering SSA by using a couple of extra segments; this can be applied in the case of the JSA as well, and for comparable geometries. The relevant quantity for evaluation of the self-flux is the flux induced in the SQUID or the JJ, and must be compared to $\Phi_{0}$ (for JSA) or to $\Phi_{0}/4$ (for SSA). We anticipate that self-flux degradation for JSA is roughly the same or less than for SSA. Therefore, we may neglect the self-flux in the evaluation of the relative performance of JSA and SSA.

When applied to the ion-damaged technology that we are using, we obtain somewhat better transfer factors for the JSA. This is primarily due to the dispersion of the JJ characteristic parameters, and more specifically to the critical current dispersion. The dispersion impact is larger on SQUIDs because of their periodic response to magnetic flux, including self-flux. Moreover, one may expect that the smaller inductance per unit length of LJJ reduces the effect of self-flux with regard to SQUIDs.

Otherwise, depending on the JJ technology, it may be advantageous to use low-transparency barriers as the bias current will be smaller, the Josephson length will be larger, and the degradation of the response for long JJs will be smaller. The technologies that are most suited to this approach are probably those technologies where the maximum achievable $I_{C}R_{N}$ product increases for lower barrier transparency, such as the ion-damaged technologies [45].

Comparisons based on the SNR values are less general, as they involve the noise level of the readout electronics $N_{A}$. Comparing the noise power only, and assuming a number of series elements $N=R_{0}/R_{1}$ to ensure good matching conditions, we evaluate

(30) $$N\cdot N_{1}+\frac{2N_{A}}{R_{0}R_{1}}\cdot {\left(R_{0}+N\cdot R_{1}\right)}^{2}=\frac{R_{0}}{\rho }\left(N_{0}L_{0}+8N_{A}L_{JJ}\right)$$

This expression shows that the noise contribution of the LJJ is independent of *N* (or equivalently of $L_{JJ}$) and that the noise contribution of the amplifier is minimized for the matching condition $N=R_{0}/R_{1}$. This indicates that the overall noise contribution might be larger for a JJ length larger than twice the length of the JJ used for the SQUIDs, i.e., 4 μm with most HTc technologies.

We plan to carry out comparisons of the experimental performance of large series arrays of SQUIDs and JJs fabricated with the ion-damaged barrier technology.

## 8. Conclusions

We have investigated the scaling of arrays of Josephson and/or SQUIDs connected in series and its limitations. We have evaluated the impact of different effects on the scaling of these arrays—Josephson parameter dispersion and self-field—and how they might be used or reduced. We propose a layout/biasing scheme for large arrays configured as meanders, which might be useful for 2D arrays or, more generally, when the bias current is large. We show that both types of arrays may be limited for wide-band application at radio frequency, essentially because of the large mismatch with the input impedance of the readout electronics. This limits the transfer factor of SQUID (resp. JJ) arrays to a few hundred Volts/Tesla (resp. around 1 kV/T). Achievable signal-to-noise ratios are degraded by the noise of the readout electronics, with a potentially higher impact for longer Josephson junctions. Possible candidate architectures to overcome these limitations are 2D arrays of JJs and/or a combination of arrays and a flux transformer/focuser.

## 9. Patents

Patent resulting from the work reported in this manuscript: FR—3096 785—B1.

## Figures and Tables

**Table 1:** Scaling of arrays of perfectly identical SQUIDs, delivering the output signal to a matched, i.e., suitably scaled, load. *M* (resp. *N*) is the number of JJs in parallel (resp. in series). The frequency bandwidth to integrate the NSD for power evaluation is assumed independent of *N* and *M*.

Arrangement	Series	Parallel	2D
Modulation $\Delta V_{N}$	*N*	1	*N*
Transfer Factor $\partial V_{N}/\partial B$	*N*	1	*N*
Impedance	*N*	$M^{-1}$	$NM^{-1}$
Output Signal Power	*N*	*M*	NM
Output NSD $S_{V}$	*N*	$M^{-1}$	$NM^{-1}$
Output Noise Power	1	1	1
Input NSD $S_{B}$	$N^{-1}$	$M^{-1}$	${\left(NM\right)}^{-1}$
SFDR	$N^{2/3}$	$M^{2/3}$	${\left(NM\right)}^{2/3}$

## Data Availability

Not applicable.

## Abbreviations

The following abbreviations are used in this manuscript:

JJ	Josephson junction
JSA	Josephson junction series array
LJJ	Long Josephson junction
NSD	Noise spectral density
SFDR	Spur-free dynamic range
SNR	Signal-to-noise ratio
SQUID	Superconducting quantum interference device
SSA	SQUID series array
WPD	Superconducting wave functions phase difference
